# Supplementary material for: ARTreeFormer: A faster attention-based autoregressive model for phylogenetic inference
Source: PLoS Comput Biol. 2025 Dec 4;21(12):e1013768. doi: 10.1371/journal.pcbi.1013768 (PMC12721553; doi:10.1371/journal.pcbi.1013768)
Supplement: S1 Text — Appendix A: Details of ARTree. Appendix B: Details of variational Bayesian phylogenetic inference. Appendix C: Proofs for the time complexity results. Appendix D: Additional experimental results. (PDF) [file pcbi.1013768.s001.pdf]

A Details of ARTree

A.1 Tree topology generating process

Let $\tau_n = (V_n, E_n)$ be a tree topology with n leaf nodes and V_n, E_n are the sets of nodes and edges respectively. Here we only discuss the modeling of unrooted tree topologies. A pre-selected order (also called the taxa order) for the leaf nodes $\mathcal{X} = \{x_1, \ldots, x_N\}$ is assumed. We first give the definition of ordinal tree topologies.

Definition 1 (Ordinal Tree Topology; Definition 1 in [17]). *Let $\mathcal{X} = \{x_1, \ldots, x_N\}$ be a set of $N(N \geq 3)$ leaf nodes. Let $\tau_n = (V_n, E_n)$ be a tree topology with $n(n \leq N)$ leaf nodes in $\mathcal{X}$. We say τ_n is an ordinal tree topology of rank n, if its leaf nodes are the first n elements of $\mathcal{X}$, i.e., $V_n \cap \mathcal{X} = \{x_1, \ldots, x_n\}$.*

The tree topology generating process is initialized by τ_3, the unique ordinal tree topology of rank 3. In the n-th step (n start from 3), assume we have an ordinal tree topology $\tau_n = (V_n, E_n)$ of rank n. To incorporate the leaf node x_{n+1} into τ_n, the following steps are taken:

1. A choice is made for an edge $e_n = (u, v) \in E_n$, which is then removed from E_n.
2. Add a new node w and two additional edges, (u, w) and (w, v) to the tree topology τ_n.
3. Add the next leaf node x_{n+1} and an additional edge (w, x_{n+1}) to the tree topology τ_n.

The above steps create an ordinal tree topology τ_{n+1} of rank $n + 1$. Repeating these steps for $n = 3, \ldots, N - 1$ leads to the eventual formation of the ordinal tree topology $\tau = \tau_N$ of rank N. The selected edges at each time step form a sequence $D = (e_3, \ldots, e_{N-1})$, which we call D a decision sequence. Here we give two main theoretical results.

Theorem 4. *The generating process $g(\cdot) : D \mapsto \tau$ is a bijection between the set of decision sequences of length $N - 3$ and the set of ordinal tree topologies of rank N.*

Theorem 5. *The time complexity of the decomposition process induced by $g^{-1}(\cdot)$ is $O(N)$.*

The bijectiveness in Theorem 4 implies that we can model the distribution $Q(\tau)$ over tree topologies by modelling $Q(D)$ over decision sequences, i.e.,

$$Q(\tau) = Q(D) = \prod_{n=3}^{N-1} Q(e_n | e_{<n}), \tag{19}$$

where $e_{<n} = (e_3, \ldots, e_{n-1})$ and $e_{<3} = \emptyset$. The conditional distribution $Q(e_n|e_{<n})$, which describes the distribution of edge decision given all the decisions made previously, is called the edge decision distribution by us.

A.2 Graph neural networks for edge decision distribution

The edge decision distribution $Q(e_n|e_{<n})$ defines the probability of adding the leaf node x_{n+1} to the edge e_n of τ_n, conditioned on all the ordinal tree topologies $(\tau_3, \ldots, \tau_n)$ generated so far. To model $Q(e_n|e_{<n})$, ARTree employs the following four modules.

Node embedding module At the n-th step of the generation process, ARTree relies on the node embedding module to assign node embeddings for the nodes of the current tree topology $\tau_n = (V_n, E_n)$. The embedding method follows [22], which first assigns one-hot encoding for the leaf nodes:

$$[f_n(x_i)]_j = \delta_{ij}, \quad 1 \leq i \leq n, \quad 1 \leq j \leq N,$$

where δ denotes the Kronecker delta function. We then obtain embeddings for the interior nodes by minimizing the Dirichlet energy, defined as

$$\ell(f_n, \tau_n) := \sum_{(u,v)\in E_n} ||f_n(u) - f_n(v)||^2.$$

This minimization process is achieved through the two-pass algorithm (Algorithm 3). Note that this process contains $(2n-6)$ sub-iterations and each sub-iteration contains a linear combination over at most 3 vectors in $\mathbb{R}^N$. The time complexity of calculating the topological node embeddings is $O(Nn)$. Finally, a linear transformation is applied to all the node embeddings to obtain the initial node features in $\mathbb{R}^d$ for message passing. It should be highlighted that the embeddings for interior nodes may vary as the number of leaf nodes n, leading to the need for time guidance in the readout module.

Algorithm 2: ARTree: an autoregressive model for phylogenetic tree topologies [17]

Input: A set $\mathcal{X} = \{x_1, \ldots, x_N\}$ of leaf nodes.
Output: An ordinal tree topology τ of rank N; the ARTree probability $Q(\tau)$ of τ.

$\tau_3 = (V_3, E_3) \leftarrow$ the unique ordinal tree topology of rank 3;
for $n = 3, \ldots, N-1$ **do**
  Let $f_n(u) = c_u f_n(\pi_u) + d_u$ where π_u is the parent of u;
  Calculate the probability vector $q_n \in \mathbb{R}^{|E_n|}$ using the current GNN model;
  Sample an edge decision e_n from Discrete$\,(q_n)$ and assume $e_n = (u, v)$;
  Create a new node w;
  $E_{n+1} \leftarrow (E_n \backslash \{e_n\}) \cup \{(u,w), (w,v), (w, x_{n+1})\}$;
  $V_{n+1} \leftarrow V_n \cup \{w, x_{n+1}\}$;
  $\tau_{n+1} \leftarrow (V_{n+1}, E_{n+1})$;
end
$\tau \leftarrow \tau_N$;
$Q(\tau) \leftarrow q_3(e_3) q_4(e_4) \cdots q_{N-1}(e_{N-1})$.

Message passing module ARTree employs iterative message passing rounds to calculate the node features, capturing the topological information of τ_n. The l-th message passing round is implemented by

$$m_n^l(u,v) = F_{\text{message}}^l(f_n^l(u), f_n^l(v)),$$
$$f_n^{l+1}(v) = F_{\text{updating}}^l\left(\{m_n^l(u,v); u \in \mathcal{N}(v)\}\right),$$

where F_{message}^l and F_{updating}^l are the message function and updating function in the l-th round, and $\mathcal{N}(v)$ is the neighborhood of the node v. The corresponding time-complexity is $O(nd^2)$ (noting that MLPs are applied to all the nodes) In particular, ARTree sets the number of message passing steps $L = 2$ and utilizes the edge convolution operator [76] for the design of F_{message}^l and F_{updating}^l.

Recurrent module To efficiently incorporate the information of previously generated tree topologies into the edge decision distribution, ARTree uses a gated recurrent unit (GRU) [77] to form the hidden states of each node. Concretely, the recurrent module is implemented by

$$h_n(v) = \mathrm{GRU}(h_{n-1}(v), f_n^L(v)),$$

where $h_n(v)$ is the hidden state of v at the n-th step in the generating process For the newly added nodes, their hidden states are initialized to zeros. This module is mainly composed of MLPs on the node/edge features, whose time complexity is $O(nd^2)$.

Readout module In the readout module, to form the edge decision distribution $Q(e_n|e_{<n})$, ARTree calculates the scalar edge feature $r_n(e) \in \mathbb{R}$ of $e = (u, v)$ using

$$\begin{aligned} p_n(e) &= F_{\text{pooling}}\left(h_n(u) + b_n, h_n(v) + b_n\right), \\ r_n(e) &= F_{\text{readout}}\left(p_n(e) + b_n\right), \end{aligned}$$

where b_n is the sinusoidal positional embedding of time step n that is widely used in Transformers [18], F_{pooling} is the pooling function implemented as 2-layer MLPs followed by an elementwise maximum operator, and F_{readout} is the readout function implemented as 2-layer MLPs with a scalar output. This module is mainly composed of MLPs on the node/edge features, whose time complexity is $O(nd^2)$. The edge decision distribution is

$$Q(\cdot|e_{<n}) \sim \text{Discrete}\left(q_n\right), \quad q_n = \text{softmax}\left(\{r_n(e)\}_{e \in E_n}\right),$$

where $q_n \in \mathbb{R}^{|E_n|}$ is a probability vector.

Let $\boldsymbol{\phi}$ be all the learnable parameters in GNNs. Then the ARTree based probability of a tree topology τ takes the form

$$Q_{\boldsymbol{\phi}}(\tau) = Q_{\boldsymbol{\phi}}(D) = \prod_{n=3}^{N-1} Q_{\boldsymbol{\phi}}(e_n|e_{<n}),$$

The whole process of ARTree for generating a tree topology is summarized in Algorithm 2.

B Details of variational Bayesian phylogenetic inference

By positing a tree topology variational distribution $Q_{\boldsymbol{\phi}}(\tau)$ and a branch length variational distribution $Q_{\boldsymbol{\psi}}(\boldsymbol{q}|\tau)$ which is conditioned on tree topologies, the variational Bayesian phylogenetic inference (VBPI) [21] approximates the phylogenetic posterior $p(\tau, \boldsymbol{q}|\boldsymbol{Y})$ in Eq (2) with $Q_{\boldsymbol{\phi},\boldsymbol{\psi}}(\tau, \boldsymbol{q}) = Q_{\boldsymbol{\phi}}(\tau)Q_{\boldsymbol{\psi}}(\boldsymbol{q}|\tau)$. To find the best approximation, VBPI maximizes the following multi-sample lower bound

$$L^K(\boldsymbol{\phi}, \boldsymbol{\psi}) = \mathbb{E}_{Q_{\boldsymbol{\phi},\boldsymbol{\psi}}(\tau^{1:K}, \boldsymbol{q}^{1:K})} \log\left(\frac{1}{K}\sum_{i=1}^{K} \frac{p(\boldsymbol{Y}|\tau^i, \boldsymbol{q}^i)p(\tau^i, \boldsymbol{q}^i)}{Q_{\boldsymbol{\phi}}(\tau^i)Q_{\boldsymbol{\psi}}(\boldsymbol{q}^i|\tau^i)}\right).$$

where $Q_{\boldsymbol{\phi},\boldsymbol{\psi}}(\tau^{1:K}, \boldsymbol{q}^{1:K}) = \prod_{i=1}^{K} Q_{\boldsymbol{\phi},\boldsymbol{\psi}}(\tau^i, \boldsymbol{q}^i)$. Compared to the single-sample lower bound, the multi-sample lower bound enables efficient variance-reduced gradient estimators and encourages exploration over the vast and multimodal tree space. However, as a large K may also reduce the signal-to-noise ratio and deteriorate the training of variational parameters [78], a moderate K is suggested [51]. In practice, the

Algorithm 3: Two-pass algorithm for topological embeddings for internal nodes [22]

Input: Tree topology $\tau_n = (V_n, E_n)$ of rank n, where $V_n = V_n^b \cup V_n^o$; Topological embeddings for the leaf nodes $\{f_n(u)|u \in V_n^b\}$.

Output: Topological embeddings for the leaf nodes $\{f_n(u)|u \in V_n^o\}$

Initialized $c_u = 0, d_u = f_n(u)|u \in V_n^b$;

for u *in the postorder traverse of* τ_n **do**

  if u *is not the root node* **then**

    Compute

$$c_u = \frac{1}{|\mathcal{N}(u)| - \sum_{v\in \mathrm{ch}(u)} c_v}, \quad d_u = \frac{\sum_{v\in \mathrm{ch}(u)} d_v}{|\mathcal{N}(u)| - \sum_{v\in \mathrm{ch}(u)} c_v}$$

    where $\mathcal{N}(u)$ is the neighborhood of u and $\mathrm{ch}(u)$ is the set of the children of u.

end

for u *in the preorder traverse of* τ_n **do**

  if u *is not the root node* **then**

    Let $f_n(u) = c_u f_n(\pi_u) + d_u$ where π_u is the parent of u.

  else

    Let $f_n(u) = \frac{\sum_{v\in \mathrm{ch}(u)} d_v}{|\mathcal{N}(u)| - \sum_{v\in \mathrm{ch}(u)} c_v}$.

  end

end

gradients of the multi-sample lower bound w.r.t the tree topology parameters $\boldsymbol{\phi}$ and the branch length parameter $\boldsymbol{\psi}$ can be estimated by the VIMCO/RWS estimator [42,79] and the reparameterization trick [44] respectively. Specifically, the gradient $\nabla_{\boldsymbol{\phi}} L^K(\boldsymbol{\phi}, \boldsymbol{\psi})$ can be expressed as

$$\nabla_{\boldsymbol{\phi}} L^K(\boldsymbol{\phi}, \boldsymbol{\psi}) = R_1 + R_2,$$

$$R_1 = \mathbb{E}_{Q_{\boldsymbol{\phi},\boldsymbol{\psi}}(\tau^{1:K}, \boldsymbol{q}^{1:K})} \nabla_{\boldsymbol{\phi}} \log \left(\frac{1}{K} \sum_{i=1}^{K} \frac{p(\boldsymbol{Y}|\tau^i, \boldsymbol{q}^i) p(\tau^i, \boldsymbol{q}^i)}{Q_{\boldsymbol{\phi}}(\tau^i) Q_{\boldsymbol{\psi}}(\boldsymbol{q}^i|\tau^i)} \right)$$

$$R_2 = \mathbb{E}_{Q_{\boldsymbol{\phi},\boldsymbol{\psi}}(\tau^{1:K}, \boldsymbol{q}^{1:K})} \sum_{i=1}^{K} \log \left(\frac{1}{K} \sum_{i=1}^{K} \frac{p(\boldsymbol{Y}|\tau^i, \boldsymbol{q}^i) p(\tau^i, \boldsymbol{q}^i)}{Q_{\boldsymbol{\phi}}(\tau^i) Q_{\boldsymbol{\psi}}(\boldsymbol{q}^i|\tau^i)} \right) \nabla_{\boldsymbol{\phi}} Q_{\boldsymbol{\phi},\boldsymbol{\psi}}(\tau^i, \boldsymbol{q}^i).$$

VIMCO considers the following expression of R_2,

$$R_2 = \mathbb{E}_{Q_{\boldsymbol{\phi},\boldsymbol{\psi}}(\tau^{1:K}, \boldsymbol{q}^{1:K})} \sum_{i=1}^{K} \left\{ \log \left(\frac{1}{K} \sum_{i=1}^{K} \frac{p(\boldsymbol{Y}|\tau^i, \boldsymbol{q}^i) p(\tau^i, \boldsymbol{q}^i)}{Q_{\boldsymbol{\phi}}(\tau^i) Q_{\boldsymbol{\psi}}(\boldsymbol{q}^i|\tau^i)} \right) - \hat{f}_i \right\} \nabla_{\boldsymbol{\phi}} Q_{\boldsymbol{\phi},\boldsymbol{\psi}}(\tau^i, \boldsymbol{q}^i)$$

where $\hat{f}_i = \log \left(\frac{1}{K-1} \sum_{j\neq i} \frac{p(\boldsymbol{Y}|\tau^j, \boldsymbol{q}^j) p(\tau^j, \boldsymbol{q}^j)}{Q_{\boldsymbol{\phi}}(\tau^j) Q_{\boldsymbol{\psi}}(\boldsymbol{q}^j|\tau^j)} \right)$ is a control variate.

The tree topology model $Q_{\boldsymbol{\phi}}(\tau)$ can be parametrized by ARTree, which enjoys unconfined support over the tree topology space. In addition to ARTree, subsplit Bayesian networks (SBNs) have long been the common choice for $Q_{\boldsymbol{\phi}}(\tau)$. In SBNs, a subset C of the leaf nodes is called a clade, and an ordered pair of two clades (C_1, C_2) is called a subsplit of C if $C_1 \cup C_2 = C$. For each internal node on a tree topology τ, it corresponds to a subsplit s determined by the descendant leaf nodes of its children. The SBNs are then parametrized by the probabilities of the root subsplit $\{p_{s_1}; s_1 \in \mathbb{S}_{\mathrm{r}}\}$ and

the probabilities of the child-parent subsplit pairs $\{p_{s|t};\ s|t \in \mathbb{S}_{\mathrm{ch|pa}}\}$. For an unrooted tree topology $\tau = (V, E)$, its SBN based probability is

$$Q_{\mathrm{sbn}}(\tau) = p_{s_r} \prod_{u \in V^o; u \neq r} p_{s_u|s_{\pi_u}},$$

where V^o is the set of internal nodes, r is the root node, π_u are the parents of u, and s_u is the subsplit assignment of the node u. As the size of $\mathbb{S}_{\mathrm{r}}$ and $\mathbb{S}_{\mathrm{ch|pa}}$ explodes combinatorially as the number of taxa increases, SBNs rely on subsplit support estimation for a tractable parameterization. The subsplit support estimation can be difficult when the phylogenetic posterior is diffuse, and makes the support of SBNs cannot span the entire tree topology space. We refer the readers to [16] and [21] for a detailed introduction to SBNs as well as their application to VBPI.

The branch length model $Q_{\boldsymbol{\psi}}(\boldsymbol{q}|\tau)$ is often taken to be a diagonal lognormal distribution, which can be parametrized using the learnable topological features [22] of τ as follows. This approach first assigns the topological node embeddings $\{f_u\}_{u \in V}$ to the nodes on τ (Algorithm 3) and then forms the node features $\{h_u\}_{u \in V}$ using message passing networks over τ. Usually, these message passing networks take the edge convolutional operator [76]. For each edge $e = (u, v)$ in τ, one can obtain the edge features using $h_e = p(h_u, h_v)$ where p is a permutation invariant function called the edge pooling. At last, the mean and standard deviation parameters for the diagonal lognormal distribution are given by

$$\mu(e, \tau) = \mathrm{MLP}^{\mu}(h_e), \quad \sigma(e, \tau) = \mathrm{MLP}^{\sigma}(h_e)$$

where MLP^{μ} and MLP^{σ} are two multi-layer perceptrons (MLPs). In the VBPI experiment in Section Variational Bayesian phylogenetic inference, the collaborative branch length models for all SBN, ARTree, and ARTreeFormer are parametrized in this way.

C Proofs of Proposition 1 and Proposition 3

Proof of Proposition 1 We discuss each component of the complexity in ARTree as follows.

- In each step, the time complexity for computing the topological node embedding is $O(Nn)$. The overall complexity of generate B topologies is $B\sum_{n=3}^{N-1} O(Nn) = O(BN^3)$. As the tree traversal $O(N)$ and autoregressive growth $O(N)$ are not vectorizable, the overall complexity becomes $O(N^2)$ in the ideal case of vectorization.
- In each step, the time complexity of the message passing module is $O(Lnd^2) + O(nkN + nkd)$. The overall complexity of generate B topologies is $B\sum_{n=3}^{N-1} O(Lnd^2) + O(nkN + nkd) = O(BLN^2d + BN^2k(N + d))$. As the autoregressive growth $O(N)$ and sequential message passing blocks $O(L)$ are not vectorizable, the overall complexity becomes $O(LN)$ in the ideal case of vectorization.

Therefore, the overall time complexity is $O(BN^3 + BLN^2d + BN^2k(N + d))$. This complexity becomes $O(N^2 + LN)$ in the ideal case of vectorization.

Proof of Proposition 3 We discuss each component of the complexity of ARTreeFormer as follows.

- In each step, the time complexity of fix-point algorithm is $O(n^2 \log_2 M_\varepsilon)$. The overall complexity of generate B topologies is $B \sum_{n=3}^{N-1} O(n^2 \log_2 M_\varepsilon) = O(BN^3 \log_2 M_\varepsilon)$. As the autoregressive growth $O(N)$ and number of iterations $O(\log_2 M_\varepsilon)$ are not vectorizable, the overall complexity becomes $O(N \log_2 M_\varepsilon)$ in the ideal case of vectorization.

- In each step, the time complexity of the message passing module is $O(nk(N+d)) + O(nd+d^2) + O(nd^2) = O(nk(N+d) + nd^2)$. The overall complexity of generate B topologies is $B \sum_{n=3}^{N-1} O(nk(N+d)+nd^2) = O(BN^2d^2 + BN^2k(N+d))$. As the autoregressive growth $O(N)$ and sequential message passing blocks $O(L)$ are not vectorizable, the overall complexity becomes $O(N)$ in the ideal case of vectorization.

Therefore, the overall time complexity is $O(BN^3 \log M_\varepsilon + BN^2d^2 + BN^2k(N+d))$. This complexity becomes $O(N(\log M_\varepsilon + 1))$ in the ideal case of vectorization.

D Additional experimental results

D.1 Additional results on tree topology density estimation

Fig 7 shows the performance of different methods on DS1. Both ARTree and ARTreeFormer provide more accurate probability estimates for the tree topologies on the two peaks of the posterior distribution, compared to SBN-EM and SBN-SGA. We see that ARTreeFormer can provide the same accurate probability estimates as ARTree, which proves the effectiveness of ARTreeFormer.

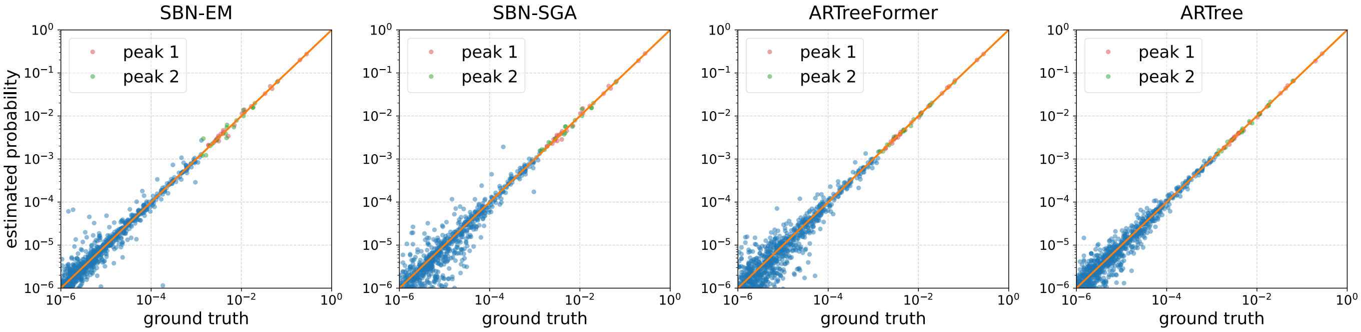

Fig 7. Performances of different methods for tree topology density estimation on DS1.

For ARTreeFormer, we also conducted an ablation study about the number of heads h and the hidden dimension d in the multi-head attention block (Table 4). For DS1-4, we train the ARTreeFormer model on the ground truth data set with a batch size of 10 and a learning rate of 0.0001, and evaluate the KL divergence towards the ground truth after 200,000 iterations. In most cases, the KL divergence gets better as the hidden dimension d increases, while it is not so sensitive to the number of heads.

D.2 Additional results on variational Bayesian phylogenetic inference

To fully demonstrate the computational burden of ARTreeFormer compared to ARTree, we report the parameter size and memory usage of ARTreeFormer and ARTree for VBPI in Table 5. We see that ARTreeFormer has less memory consumption compared

Table 4. KL divergences (↓) to the ground truth obtained by ARTreeFormer with different hyper-parameters on TDE.

Hyper-parameters	$h = 2, d = 100$	$h = 4, d = 100$	$h = 4, d = 200$	$h = 8, d = 200$
DS1	0.0058	0.0060	0.0039	0.0039
DS2	0.0002	0.0002	0.0003	0.0002
DS3	0.0058	0.0052	0.0055	0.0054
DS4	0.0097	0.0101	0.0069	0.0071

to ARTree, because ARTreeFormer does not need to update all the node features on the tree topology, in analogy with the shorter sequence length in language modeling.

Table 5. The parameter size and memory usage of ARTreeFormer and ARTree for VBPI.

Data set	DS1	DS2	DS3	DS4	DS5	DS6	DS7	DS8
ARTree (learnable parameter size)	194K	195K	197K	199K	203K	203K	207K	209K
ARTreeFormer (learnable parameter size)	215K	216K	216K	217K	218K	218K	219K	219K
ARTree (memory)	1143MB	1395MB	1376MB	1680MB	1817MB	1698MB	2070MB	2148MB
ARTreeFormer (memory)	556MB	577MB	630MB	690MB	798MB	794MB	896MB	1044MB
